# Long-lasting insecticidal net source, ownership and use in the context of universal coverage: a household survey in eastern Rwanda

**DOI:** 10.1186/s12936-015-0915-9

**Published:** 2015-10-06

**Authors:** Fredrick Kateera, Chantal M. Ingabire, Emmanuel Hakizimana, Alexis Rulisa, Parfait Karinda, Martin P. Grobusch, Leon Mutesa, Michèle van Vugt, Petra F. Mens

**Affiliations:** Medical Research Centre Division, Rwanda Biomedical Centre, PO Box 7162, Kigali, Rwanda; Division of Internal Medicine, Department of Infectious Diseases, Centre of Tropical Medicine and Travel Medicine, Academic Medical Centre, Meibergdreef 9, 1100 DE Amsterdam, The Netherlands; Malaria and Other Parasitic Diseases Division, Rwanda Biomedical Centre, Kigali, Rwanda; Department of Cultural Anthropology and Development Studies and Centre for International Development Issues, Radboud University, 6500 HE Nijmegen, The Netherlands; College of Medicine and Health Sciences, University of Rwanda, PO Box 3286, Kigali, Rwanda; Royal Tropical Institute/Koninklijk Instituut voor de Tropen, KIT Biomedical Research, Meibergdreef 39, 1105 AZ Amsterdam, The Netherlands

**Keywords:** Malaria, Households, Long-lasting insecticidal nets, Ownership, Use, Rwanda

## Abstract

**Background:**

Universal long-lasting insecticidal net (LLIN) coverage (ULC) has reduced malaria morbidity and mortality across Africa. Although information is available on bed net use in specific groups, such as pregnant women and children under 5 years, there is paucity of data on their use among the general population. Bed net source, ownership and determinants of use among individuals from households in an eastern Rwanda community 8 months after a ULC were characterized.

**Methods:**

Using household-based, interviewer-administered questionnaires and interviewer-direct observations, data on bed net source, ownership and key determinants of net use, including demographics, socio-economic status indicators, house structure characteristics, as well as of bed net quantity, type and integrity, were collected from 1400 randomly selected households. Univariate and mixed effects logistic regression modelling was done to assess for determinants of bed net use.

**Results:**

A total of 1410 households and 6598 individuals were included in the study. Overall, the proportion of households with at least one net was 92 % while bed net usage was reported among 72 % of household members. Of the households surveyed, a total ownership of 2768 nets was reported, of which about 96 % were reportedly LLINs received from the ULC. By interviewer-physical observation, 88 % of the nets owned were of the LLIN type with the remaining 12 % did not carry any mark to enable type recognition. The odds of bed net use were significantly lower among males and individuals: from households of low socio-economic status, from households with <two bed nets, from households reporting use of ≥two sleeping spaces, and those reporting to have not slept on a bed.

**Conclusion:**

In this study, despite high a bed net coverage, over 25 % of members reported not to have slept under a bed net the night before the survey. Males were particularly less likely to use bed nets even where nets were available. Household socio-economic status, number of bed nets and type and number of sleeping spaces were key determinants of bed net use. To maximize impact of ULC, strategies that target males as well as those that ensure ITN coverage for all, address barriers to feasible and convenient bed net use including covering over all sleeping space types, and provide net hanging supports, are needed.

## Background

Insecticide-treated bed nets (ITNs) are a cornerstone of malaria control in sub-Saharan Africa [1, 2]. The World Health Organization (WHO) recommends universal access to, and use of long-lasting insecticidal nets (LLINs) for all individuals at risk of malaria so as to optimize ITN effectiveness [3]. ITNs act by placing a physical barrier between the mosquito and humans and through the repellent toxic effects of the ITN-impregnated insecticides. ITNs have been shown to reduce malaria burden at both individual and community level leading to decreased morbidity, mortality and overall transmission potential [1, 4, 5]. With ITNs also shown to be the most cost-effective intervention in reducing malaria transmission [6], universal long-lasting insecticidal net coverage (ULC) has been recommended and is now widely implemented as a key intervention in malaria control efforts [7].

The impact of LLIN scale-up on reducing malaria burden has been observed in Rwanda [8]. With financial support mainly from The Global Fund to Fight AIDS, Tuberculosis and Malaria and the President’s Malaria Initiative, Rwanda achieved ULC—defined as a reported household ownership of at least one bed net per two individuals, as early as February 2011 [9]. However, despite the observed initial decline in health facility-recorded malaria cases and deaths following LLIN scale-up in Rwanda, increases in malaria burden continue to be reported [8, 10, 11]. While the resurgence in 2009 was mainly attributed to a reduced effectiveness of LLINs due to delays in provision of new nets at a time when the effectiveness of the previously provided LLINs was waning [10], later resurgence may have been partly associated with the reported deployment of bed nets with sub-optimal concentrations of insecticide [11]. However, although the reasons for the resurgence were not systematically characterized, continued scale-up and use of LLINs is needed if gains made in malaria burden decline in the past are to be sustained [10]. To achieve and maintain ULC, Rwanda adopted the WHO’s recommendations for high malaria burden countries of using multiple distribution channels, including free ULCs that are supplemented by continuous LLIN distributions through programmes such as antenatal care (ANC) and immunization services for pregnant women and infants, respectively [3, 9].

A key determinant of ITN impact is bed net use, with previous studies showing disparities between bed net ownership and use [12–17]. One such determinant of bed net use is seasonality. While higher net use has been reported more in the rainy season due to the associated high mosquito density, lower net use has been associated with hot dry months due to heat-related discomfort [12–14]. Other previously reported determinants of net use include number of nets owned per household, sex: with women more likely to use nets [15], age [15], head of household (HoH) education levels, disruptive sleeping arrangements [16], and net misuse such as bed nets being used for activities in agriculture and fishing [17]. Hitherto, studies on bed net use have mostly focused on children <5 years and pregnant women, two groups preferentially targeted for net coverage in the past because of their high malaria risk. There is limited and inconclusive data on ITN effectiveness under routine field settings after ULC targeting of all age and gender groups. Understanding these household-level bed net use patterns is needed to inform malaria control programmes on how to optimize bed net public health impact. Here, a community-based evaluation of bed net source, ownership and determinants of use was conducted 8 months after ULC.

## Methods

### Study area description and malaria risk

This cross-sectional survey was conducted among a representative sample of households randomly selected from 35 villages of a rural, predominantly agricultural, Ruhuha sector of Bugesera District in the eastern province of Rwanda from November 2014 to January 2015. Rwanda is broadly divided into four malaria ecologic zones based on altitude, climate, level of transmission, and disease vector prevalence [18]. Topographically, malaria transmission is considered meso-endemic in the plain regions of eastern and southern provinces while being epidemic-prone in the high plateau and hill settings of northern and western provinces, respectively [18]. Ruhuha sector is a rural agricultural community that is located in the high malaria transmission zone. The main malaria control interventions used in the study area include ULC, indoor residual spraying (IRS) with insecticide and use of artemisinin combination therapy (ACT).

### Study population and eligibility criteria

This study is part of a larger project that aims to use an integrated (biomedical, anthropological, entomological, and economical), community-based approach targeting reduction of malaria transmission at household level [19]. A sample of 1400 households was randomly selected from a sampling frame of 4522 sector households generated 2 months prior to the survey as part of an enumeration exercise conducted while planning for IRS exercise for the sector. To identify the randomly selected households for study inclusion, study team members visited a particular village and identified the households by the named HoH on the enumeration sheet. This study and the associated follow-up procedures were then introduced to the HoH or their spouses who were then requested to provide a written informed consent before enrolment. Data on household members aged >6 months and who had spent the night prior to the survey at the household were collected. A household was defined as any unit headed by a male or female with his/her dependents and/or spouse who shared a cooking pot/common eating-place.

### Study questionnaire and variables definitions

A pre-coded questionnaire, that was largely adapted from the standard malaria indicator survey (MIS) and the demographic health survey (DHS), was administered to the HoH or their spouse [20]. Data on demographics (age, sex, education level, occupation, and marital status), household socio-economic status (SES) indicators (including ownership of land and animals, main sources of household amenities (including lighting, cooking and drinking water), ownership of items (such as telephone, television, refrigerator, bicycle and radio), house structural features (such as type of material used to construct house wall and floor), malaria prevention knowledge and practices, bed net characteristics (of ownership, source and use), and IRS activity within 12 months prior to survey, were collected. In addition, a spot check was performed to verify bed net number, brand, shape and integrity (having holes or no holes). A bed net was classified as having holes if it had any finger-sized hole or larger. In this study, three degrees of severity of net deterioration including finger size, fist size and head size were assessed for.

### Data collection

Field workers were trained for 10 days on key survey aspects of study objectives, variable data to be collected and question intent. Additionally, classroom role-plays and piloting of questionnaires were conducted, with daily feedback reviews conducted to ensure consistency of translation and appropriateness of the wording in the local language (*Kinyarwanda*). Although the questionnaire was developed in English and data captured onto an English language electronic format, both the training and data collection exercise were conducted using paper-based questionnaires that were translated into *Kinyarwanda*. The electronic format questionnaire was developed using Open Data Kit (ODK) Collect set-up [21]. ODK is an open-source suite of tools that include ODK Collect, an android-based mobile client that acts as the interface between the user and the underlying form used to collect data [21]. The collected data were then electronically uploaded onto a central server and later exported into Microsoft Excel 2007 version (Microsoft Corp) for further analysis.

### Bed net distribution

Between January 2012 and December 2014, bed nets were distributed within the study area using multiple channels, including a ULC targeting the general population, mass distribution of bed nets for all children aged <5 years, and continuous distribution through ANC and immunization services. Prior to the ULC, community health care workers (CHWs) enumerated each household for type and number of sleeping spaces and number of bed nets available. Among the general population, 5600 LLINs were distributed in May 2012 and an additional 4550 distributed in May 2013 to achieve complete coverage of all sleeping spaces. However, following these two rounds of net distribution, the LLIN brand (Netprotect^®^) provided was later confirmed to be impregnated with sub-optimal amounts of the insecticide [11]. This led to a replacement exercise conducted in March 2014 where 10,150 LLINs were distributed to replace the sub-standard LLINs (Mukamana, pers comm). Concurrently, three supplementary distribution campaigns were run in which 3283 LLINs were distributed to cover children aged <5 years in 2012, 540 LLINs distributed to pregnant women through the ANC between 2012 and 2014, and 1295 LLINs distributed through the immunization service to cover infants.

### Ethical approval

Study protocols received ethical and scientific approval from the National Health Research Committee (NHRC) and the Rwanda National Ethics Committee (No. 20/RNEC/2015), Kigali, Rwanda.

### Statistical analysis

Data analysis was performed using STATA version 13.0 (STATA Corp., College Station, TX, USA) software. Descriptive statistics of frequencies, proportions, cross tabulations with crude Pearson’s Chi square tests between outcome and dependent variables were performed. The primary outcome was bed net use—defined as a reported history of sleeping under a bed net the night before the survey. Independent covariates reported by other studies as associated with net use, including but not limited to, age, sex, HoH education level at individual level and number of ITNs, number of residents per household and SES levels at household level were analysed individually for an association with bed net use. All variables that showed evidence for a possible association with bed net use (*p* value < 0.1) were then included in the final mixed effect logistic regression model. This model was chosen to ensure adjustment for individual intra-cluster and household inter-cluster correlation. The risk of no bed net use under final multivariate model was considered significant for variables with an effect with a *P* value ≤0.05 based on Wald tests.

### Generating household-level socio-economic status (SES) scores

Measures of household wealth can be reflected by income, consumption or expenditure-related indicator information. To generate household-level SES scores using principal component analysis (PCA) as described elsewhere [22, 23], 17 indicators were used: (1) any household member ownership of television (yes/no), radio (yes/no), bicycle (yes/no), and telephone (yes/no); (2) HoH ownership of house lived in (yes, no—pay rent, no—use without paying rent); (3) type of sources for: (a) lighting (electricity, kerosene, oil, gas or paraffin lamps, solar, firewood, candles/battery/flash lights, others), (b) cooking (electricity, biogas/LPG/natural gas, paraffin, charcoal, firewood/straw, others), (c) domestic water (private connection to pipeline, public well, borehole, harvested rain water, river, stream, lake, or other surface water, public tap, public tap/standpipe, bottled water, others), and (d) toilet (flash toilet, pit latrine, ventilated improved pit (VIP) latrine, no facility/bush/field); (4) material used to construct: (a) house walls (burnt bricks, cement/concrete blocks, adobe/un-burnt bricks, mud/poles, others) and (b) house floors (carpet, parquet, polished wood, mosaic or tiles, cement/concrete, bricks, clay/earth, dung/sand); (5) HoH enrolment into any health insurance (yes/no); (6) HoH membership into an economic group (yes/no); (7) ability of HoH to save any money in past 3 months (yes/no); (8) ability of HoH to pay for medical services (yes/no) and ability of HoH to pay for medications prescribed (yes/no); and, (9) highest level of HoH education (none, primary, secondary, tertiary). Other SES indicator variables for whom data was collected but that had a frequency of <1 % were omitted due to their low ability to differentiate between households. The PCA derived scores were considered as weight (eigenvectors of the correlation matrix) for each variable and the sum of the weights per household considered as the household level SES score. The scores were then ranked in terciles with the highest 33 % of household considered high SES, the lowest 33 % as low SES and the rest as middle SES [23].

## Results

### Baseline household characteristics

Of the pre-selected 1410 households, six were unoccupied as household members were reported to have moved out of the sector, six HoH did not provide study consent, ten did not have an eligible person to be interviewed, and 23 households could not be identified because the residents did not know the named HoH. For all omitted households, the nearest household in the same village was identified as a replacement.

Data collected covered 1400 households and 6598 individuals of whom the mean (±SD) age was 22.9 (±18.3), 3282 (53.3 %) were female, 582 (9.0 %) were children <5 years. The mean (±SD) number of household members was 4.7 (±1.9) (Table 1).

**Table 1:** Household (N = 1410) socio-demographic and house structural features, Ruhuha sector, Rwanda, 2015

Variable name	Variable groups	Frequency, n (%)
Sex of head of household (HoH)	Male	1021 (72.4)
	Female	389 (27.6)
Age of HoH in years	Mean (±SD)	44.7 (±1 4.5)^a^
Highest educational level attained by HoH	None	415 (29.4)
	Primary school	790 (56.0)
	Post primary/vocational	36 (2.6)
	Secondary or higher	169 (12.0)
Marital status of HoH	Never married	46 (3.3)
	Married	654 (46.4)
	Living together	319 (22.6)
	Separated/divorced	108 (7.6)
	Widowed	283 (20.1)
Household (HH) member size	Mean (±SD)	4.69 (±1.9)^a^
Proportion of HoH with no formal education	None	415 (29.4)
HH socio-economic status (SES) score	Low	470 (33.4)
	Middle	470 (33.4)
	High	468 (33.2)
Does the HH own the house currently lived in?	Yes	1250 (88.8)
	No	158 (11.2)
Type of sleeping spaces used in HH	Beds	1819 (62.9)
	Floor	1075 (37.1)
Average number sleeping spaces in HH visited	Mean (±SD)	2.17 (±0.9)^a^
Number of sleeping spaces per HH	1	294 (20.9)
	2	697 (49.5)
	3	339 (24.0)
	≥4	79 (5.6)
Average number of rooms in household visited	Mean (±SD)^a^	2.96 (±1.2)
Number of rooms in house lived in	1	141 (10.1)
	2	250 (17.7)
	3	738 (52.4)
	4	175 (12.4)
	≥5	105 (7.4)
Number of windows in the house lived in	0–2	419 (29.7)
	≥3	991 (70.1)
Did the house have a ceiling?	Yes	101 (7.1)

^a^Standard deviations

The majority (70.9 %) of households had permanent (bricks/cement) wall structures whilst the others households had walls made of temporary (mud and poles) materials. Only 7.1 % and 8.9 % of the households had permanent structured walls and ceilings, structures beneath the roof where bed nets are usually hung, respectively (Table 1).

Two types of sleeping space were identified including a raised up platform (bed) and floor-based spaces. The majority (although not quantified in this study) of the floor-based spaces (although not quantified in this study) were fixed and consistently used spaces, where a mattress or other beddings were placed on the floor. The majority (70.3 %) of the households reported using ≥two sleeping places while the commonest type of sleeping space used was a bed (62.9 %) (Table 1). By households SES levels, the proportions of bed net use among individuals of low, middle and high SES households were 56.5, 64.3 and 65.4 %, respectively.

### Bed net ownership, source and integrity

A majority of 91.7 % households reported owning at least one bed net with a total ownership of 2769 nets reported (1.96 nets/HH). Of the total nets, 86.2 % were reported not frequently in use, with commonest reasons for not using these nets including not needed or lack of where to hang them or not easy to use them due to shape and/or distance between point of hanging and levels of bedding. The majority (95.6 %) of nets were received through the ULC with the others either purchased (n = 19), or received from family members (n = 28) or received from the ANC and immunization clinics (n = 75). By on-spot study interviewer observations on net integrity and brand type, 344 (12.9 %) of the nets had at least one hole (of any size) while a total of 2281 (87.9 %) nets were identified as LLINs (Tuzanet, Mamanet and PermaNet^®^) while the remaining 12.1 % were found not to carry any mark to enable brand/type recognition, respectively. Details of net source, ownership and integrity are reported in Table 2.

**Table 2:** Bed net source, ownership and use, Ruhuha Sector, Rwanda, 2015

Variable	Variable groups	Frequency, n (%)
Bed net ownership per HH	Number of HHs with at least one bed net	1292 (91.7)
	Number of HHs without any bed net	118 (8.3)
Bed net source	From government through mass LLIN campaigns	2647 (95.6)
	From government through antenatal care and immunization clinics	75 (2.7)
	Privately purchased	19 (0.7)
	Provided for by family/relatives	28 (1.0)
Number of bed nets ownership per HH	1	326 (23.3)
	2	595 (42.6)
	3	275 (11.7)
	≥4	96 (6.8)
	No responses	104 (7.4)
Total number of nets owned in HHs visited	N	2768
Number of nets used night before the survey	n (%)	2386 (86.2)
Mean number of bed nets per HH	Mean (±SD)	2.1 (±0.3)^a^
Mean number of sleeping spaces (N = 6603)	Mean (±SD)	2.1 (±0.9)^a^
Ratio of bed nets per sleeping space	Mean (±SD)	1.0 (±0.4)^a^
Number of persons who slept under net	n (%)	3,525 (72.3)
Number of bed net used by sex	Female	1895 (72.9)
	Male	1630 (71.6)
Number of bed net used by age group	<5 years	432 (74.9)
	5–15 years	810 (68.9)
	>15 years	3523 (73.1)
Number of bed net used by sleeping space type	Slept on beds	1387 (81.6)
	Slept with no beds	576 (64.2)
Bed net integrity: presence and size of holes	Number of nets with no hole	2425 (87.7)
	Number of nets with at least one finger size hole	200 (7.2)
	Number of nets with at least one hand size hole	81 (2.9)
	Number of nets with at least one head size hole	62 (2.2)

^a^Standard deviations

### Individual net use

Overall, 72.3 % individuals reported use of a bed net the night before survey with females (72.9 %) reporting a slightly higher proportion compared to males (71.6 %). By age group, 5–15 year olds reported a lower net use (68.9 %) relative to children <5 years (74.9 %) and persons aged ≥16 years (73.1 %), respectively (Table 2). Notably, net use was much higher for individuals who slept on a bed (81.6 %) compared to 64.2 % those who reported sleeping on a mattress placed on the floor (Table 2). In 53.9 % of the household, at least one person did not sleep on a permanent bed but used a sleeping place laid on the ground. However, because individual level data on type of sleeping space used night before survey were not collected, it was impossible to directly assess the association between bed net use and type of sleeping space. In total, 13.8 % of the nets were reportedly not in frequent use although owned, with the commonest reasons as reported by interviewees but not verified by study team, for not using a bed net being the discomfort associated the hot season period (38.9 %), infestation with bed bugs that was associated with use of bed nets (18.3 %), no particular reason (6.6 %), net being damaged (2.6 %), and no need to use a bed net as after a recent IRS activity (2.4 %).

### Determinants of net use

Based on univariate analysis, a strong relation was found between net use and house structure characteristics. Persons living in houses with walls made of brick/cement blocks had a 2.4-fold higher odds of net use relative to persons living in houses with walls were made of mud and poles. In addition, the number of doors and windows a house had influenced bed net use by univariate analysis. Individuals from houses with ≥two doors and those from houses with ≥three windows had six- and fourfold more odds of bed net use than individuals with <two and <three doors and windows, respectively. Univariate results are shown in Table 3. However, for both number of doors and number of windows variables, this effect did not retain significance after adjusting for all the other determinants in the final model.

**Table 3:** Logistic regression analysis of determinants of bed net use among individuals (n = 6598) from households with ≥one net Ruhuha sector, Rwanda, 2015

Variable	Variable Sub-group	Univariate OR (95 % CI), P value	Multivariate OR (95 % CI), P value
Sex	Female (reference)	–	–
	Male	0.57 (0.44–0.74), <0.0001	0.42 (0.28–0.64), <0.0001
Age group of all HH members in years	0–5 years (reference)	–	–
	6–15	0.38 (0.23–0.63), <0.0001	0.197 (0.01–3.01), 0.243
	>15	0.86 (0.54–1.36), 0.510	0.35 (0.03–4.69), 0.431
Age group of HoH in years.	18–30 (reference)	–	–
	31–55	0.93 (0.42–2.07), 0.852	–
	56+	0.36 (0.14–0.96), 0.042	–
Education level of HoH	Any vs. none (reference)	2.53 (1.29–4.96), 0.007	0.86 (0.38–1.94), 0.720
Household SES score level	Low (reference)	–	–
	Middle	1.95 (0.92–4.12), 0.079	2.26 (1.06–4.82), <0.0001
	Upper	1.88 (0.89–3.97), 0.099	2.92 (1.31– 6.46), < 0.0001
Number of members per HH	1–3 (reference)	–	–
	4–6	0.38 (0.19–0.76), 0.006	0.73 (0.33–1.62), 0.432
	7+	0.68 (0.27–1.67), 0.398	1.86 (0.59–5.89), 0.291
Slept on a bed last night?	Yes vs. no (reference)	5.24 (3.15–8.72), <0.0001	3.01 (1.79–5.08), <0.0001
Number of sleeping space used in HH	1 (reference)	–	–
	2	1.02 (0.86–1.22), 0.790	0.34 (0.12–0.99), 0.048
	3	1.32 (1.08–1.61), 0.007	0.11 (0.03–0.40), 0.001
	4+	1.67 (1.23–2.27), <0.0001	0.07 (0.01–0.49), 0.007
Does ITN have any holes?	Yes vs. no (reference)	0.26 (0.11–0.58), 0.001	0.53 (0.23–1.18), 0.119
Number of rooms per HH	1 (reference)	–	–
	2	14.49 (3.30–63.64), <0.0001	2.43 (0.60–9.84), 0.214
	3+	22.91 (5.96–88.01), <0.0001	2.14 (0.49–9.35), 0.313
Number of doors per HH	1 (reference)		–
	2	5.26 (2.28–12.11), <0.0001	2.42 (0.84–6.98), 0.102
	3+	8.55 (2.15–34.02), 0.002	1.22 (0.23–6.64), 0.817
Number of windows per HH	1 (reference)	–	–
	2	3.44 (0.70–16.83), 0.127	3.32 (0.82–13.42), 0.092
	3	10.74 (2.41–47.79), 0.002	1.81 (0.45–7.28), 0.406
	4+	6.74 (1.24–36.72), 0.027	1.11 (0.19–6.38), 0.908
Number of bed nets used per HH	1 (reference)	–	–
	2	7.10 (4.08–12.33), <0.0001	4.72 (2.08–10.72), <0.0001
	3	12.18 (5.88–25.23), <0.0001	16.83 (5.42–52.24), <0.0001
	4+	40.32 (8.11–200.51), <0.0001	110.15 (11.99–1012.17), <0.0001

*CI* confidence interval

By univariate analysis, nets with holes were significantly less used than nets without holes (OR = 0.26, *P* = 0.001). However, this significance was not sustained after adjusting for other variables in the final multivariate model (Table 3). Variables, including a reported history of a family member experiencing a febrile illness in the past 3 months, IRS application, bed net age, HoH education, household size, houses with ceilings or houses having eaves, did not influence net use in this study.

In the final multivariate model, only five of the 13 explanatory variables including sex, household level SES level, type and number of sleeping arrangements and the number of bed nets owned showed significantly effect on bed net use (Table 3). Males showed 0.4 [95 % confidence interval (CI): 0.28–0.64] times lower odds of sleeping under a net compared to females. Also, individuals living in households of middle/high SES showed twofold higher odds of net use compared to those living in household low SES household.

Sleeping on a bed was associated with three-fold higher odds of net use (95 % CI: 1.79–5.08) compared to not sleeping on a bed. Although a reported ownership of ≥two ITNs on the night before the survey was associated with higher odds of net use in general, bed net use was also influenced by the number of sleeping places in a house. Persons from households that reported using ≥two sleeping spaces the night before the survey were associated with higher odds of net use compared to household that had only one sleeping space. Multivariate results are shown in Table 3.

## Discussion

This study demonstrated a 92 % household ownership with at least one net and a 72 % bed nets use among 1400 households visited. Particularly among men, and in households of the low SES group with ≥two sleeping places, where individuals reported not sleeping on a bed, with a reported ownership of only one bed net, lower odds of net use were observed. Also, higher odds of net use with increasing number of nets in household were observed.

Comparable to the 92 % household bed net ownership in this study, high coverage rates have been reported elsewhere following UCL, including Sierra Leone (87.6 %), Togo (96.7 %) and Ethiopia (91.0 %) [24–26]. Similarly as shown in this study, bed net use in these three settings was lower than bed net coverage, varying from 65.0 % in Ethiopia to 68.3 % in Togo and 76.5 % in Sierra Leone. This finding highlights a major need to supplement ULC with appropriate effective strategies that promote bed net use.

As observed in this study, previous studies have shown that females were more likely to use bed nets relative to males [27–29]. A possible reason for the sex disparity in net use could be the traditionally high focus on promoting net use among females through health centres and ANC-based campaigns to target reduction of malaria risk for vulnerable pregnant women. This focus may have spilled over into higher rates of net use among females even in settings of ULC and in spite of the observed lower likelihood of net use amongst men. However, the specific reasons for low rates of net use among men were not explored in this study. Characterizing these reasons is key to identifying implementation gaps and targeting strategies towards promoting net use specifically among men.

In this study, individuals who reported not to have slept on a bed had lower odds of net use compared to those who slept on beds. In one study in Kenya conducted before and after a ULC, lower odds by 0.24 and 0.31-fold decrease among individuals who reported sleeping on the floor compared to those who slept on a bed was observed [30]. In this same study, sleeping on the floor was almost fully associated with not using a net [30]. Possible reasons for lower compliance to bed net use among those not sleeping on a bed range from practical house structural challenges, including difficulty in spreading a net over a sleeping material or a mattress, lack of a suitable structure for net hanging and disruptive sleeping arrangements that complicate ease of bed net use [16, 31]. Although not studied here, it is plausible that bed net use is particularly difficult among those who did not sleep on a bed as the sleeping spaces are generally larger, irregular and much further from the point of net hanging and hence less amenable to feasible bed net use. In this study, 93 % of the houses visited had no ceiling, structures onto which nets are usually hung. It is plausible that lack of a place to hang or a need to improvise, such as by tying a long string from wall to wall onto which a net can be secured and as well as difficulty in securing net around floor-based sleeping arrangements, are some reasons for reduced likelihood of bed net use. Bed net hanging increases likelihood of bed net use [32]. Further characterization of feasibility of bed net hanging and convenience of net use among those who do not sleep on a bed is needed to promote bed net use in this group. .

Households with ≥two sleeping spaces were associated with lower odds of net use. Given the bed net to household ratio of almost 2:1 (2769 bed nets for 1410 households) in the study area, it is likely, although not specifically assessed in this study, that households with more sleeping places did not have enough bed nets to cover each sleeping space. In this study, a progressive increase in odds of net use proportional to number of used nets the night before the survey was observed. Comparable findings to this study have been reported elsewhere. In Ethiopia, a household level net density of >one net per two people was associated with a fivefold (in 2006) and a twofold (in 2007) higher net use when compared to households with net densities of <one net per two persons [32]. In Sierra Leone, a ULC was associated with a 137 % increase in bed net use within 6 months [24]. In Uganda, following a ULC, LLIN availability was the only determinant of bed net use [31]. This is plausible in this study area where there is a discrepancy between mean of household members size (4.7) and mean number of available nets (2.1), which is lower than the target of having one net per two household occupants. Therefore, since a greater intra-household access to an ITN is a strong determinant of net use, efforts to increase access to enough bed nets, particularly in households with many members, is required. To further increase net use among all age and gender sub-groups, net distribution campaigns should target coverage of at least of all sleeping spaces and ideally coverage of two nets per three persons or even one net per person.

Medium and high SES group households were associated with higher odds of bed net use in this study. Similar to findings in this study, higher net use amongst households with higher SES has been reported previously in Uganda [33], and in Ethiopia [34]. A possible reason for this observation may be that individuals from medium and high SES households have better information on access and capacity to buy supplementary LLINs and hence are more likely to use bed nets. Interestingly, associations between household SES and net use have been reported with mixed outcomes. In a smaller, prospective, hospital-based study in Nigeria, household SES did not influence bed net use [35]. In contrast, Auta et al. in a study based on data extracted from a demographic and health survey exercise in Nigeria found higher rates of net use among individuals from the lowest wealth quintile [36]. In the latter study, higher rates of net used was associated partly with a higher perception of malaria risk in the poorest settings that may have arisen from more concerted public health campaigns conducted in the area [36]. On the contrary, higher SES group households may have greater access to more nets or more favourable factors that enhance adherence to net use.

The methodology employed and study findings had major strengths. Interviewer-spot checks in assessing bed net ownership, integrity and brand as well as verifying house structural feature characteristics limited potential recall and socio-desirability bias. In addition, both the interview questions used that were adapted from the standardized MIS and DHS tools and the quantitative analysis employed served to optimize study accuracy. This study evaluated for a key outcome of bed net use in a setting of high net coverage and hence provided rich data on the effectiveness of a UCL in a real community setting. The methodology used in this study had some limitations. Firstly, the decision to replace the 35 non-enrolled houses randomly selected households with nearest neighbour households may have had an effect on representativeness of the study findings. This most likely did no affect accuracy of study findings given that the proportion of replaced households was <2.5 % of total sample size. Secondly, this being a cross-sectional survey, study findings may be confounded by unmeasured factors, not be suitable for drawing causal inferences, and not be appropriate for showing how net ownership and use may vary over time. A possible social desirability bias of over-reporting may also have influenced rates of reported net ownership and use. In addition, the study area has had many bed net campaigns that may have positively influenced knowledge and attitudes on malaria prevention and in particular, led to higher rates of net use. Study findings may not be representative of low malaria endemic settings with low bed net coverage and limited awareness of bed net use. Given that this survey covered a relatively limited area, findings may not be generalizable to the entire country and more so in settings when ULC were not conducted.

## Conclusion

Bed net ownership of ≥one net among households visited and a reported individual use among households members of 92 and 72 % was observed in the study area. This study confirmed that males in general and individuals from households of low SES, with one or more nets, where ≥two sleeping spaces are used, and those who slept on the floor relative to those who used beds, were less likely to use a net. Supplementary to LLIN scale-up campaigns, strategies to promote bed net use, particularly among males and houses with structural features that prevent mosquito entry and those that adapt bed net feasibility towards ease of use in groups such as those who do not sleep on a bed, are needed. Also, further studies on feasibility and cost-effectiveness research of ULC, as well as in-depth anthropological studies characterizing bed net use patterns, including reasons for lower net use among males, perceptions on bed net hanging, net characteristics that may lead to reduced bed net use, such as dirtiness, smells, shape, and colour and challenges of net use among those do not sleep on beds, would provide rich contextual data to inform future strategies aimed at improved net use.
